# Worms and Germs, Drink and Dementia: US Health, Society, and Policy in the Early 20th Century

**Published:** 2008-09-15

**Authors:** Lynne S Wilcox

**Affiliations:** Centers for Disease Control and Prevention

## Introduction

Case study and narrative are essential and complementary ways to understand history: case study dissects historical events into their logical elements, whereas narrative connects us to the sum of these elements, which may be startlingly different from the outcomes predicted by logic. The historical stories in this report describe selected US public health events from 1900 to 1932 and link these experiences to modern leadership challenges in public health policy.

In 1991 Richmond and Kotelchuck proposed a model for public policy that can be represented by a 3-circle Venn diagram (1,2). The circles represent scientific evidence (sound data regarding the problem), social infrastructure (systems in place to support a solution), and political will (public support and resources to achieve the solution). In the center of the diagram, where these 3 sectors overlap, is public policy (Figure).

**Figure.**
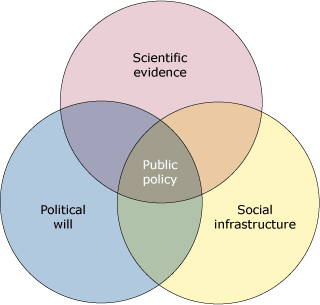
Model of Public Policy in Relation to Scientific Evidence, Social Infrastructure, and Political Will

The stories in this article illustrate how the sectors of this model came together, for better or worse, in the early 20th century in the United States. Hookworms in the South (worms), cholera infantum in New York City (germs), adulterated alcohol in the Midwest (drink), and pellagra in the Mississippi Delta (dementia) are examined in terms of how diseases and their victims were viewed, diagnosed, and treated at that time. Medical progress also affected these events, but the history of medicine is not my primary focus.

For public health professionals, the science and infrastructure sectors of the Richmond-Kotelchuck model are often where we feel most comfortable and best trained. The descriptions of Southeastern hookworm health education programs, infant milk distribution systems, adulterated alcohol investigations, and Goldberger's pellagra research all resonate with our experience and expertise.

Political will is another matter. Why did Southerners refuse to believe hookworm and pellagra existed in their states? Why did a 20-year partnership of physicians and social progressives collapse and scatter the resources for infant health programs? Why was there little outrage when tens of thousands of Americans were paralyzed by a poisoned patent medicine? Why did public health leaders play only small roles in these widespread health events?

It can be argued that most of our failures are in the arena of political will. Health policy courses appear in public health education curricula, but even experienced public health leaders trip on the complexity of political interactions. Public health practitioners in government agencies may be further restricted because of our responsibilities as public servants to remain apolitical. Our efforts to affect public policy are often frustrated.

Health is inextricably woven into the geography, warfare, politics, poverty, population, infrastructure, economy, and prejudices of a region. As Szreter has noted:

The problem, of course, with emphasizing the importance of politics, the conflict of ideas, the role of the state, conditions of citizenship, local government structures and services, civil institutions and social capital in accounting for the relationship between public health and social and economic change is that this makes for a devilishly complicated story (3).

We usually analyze history by teasing out each of these elements for in-depth case study. The Richmond-Kotelchuck model offers a more integrated approach to recognizing the missing elements for sound policy. This article provides such analyses; it also seeks to do the reverse — to bring all the elements into narratives that illustrate how public policy is conceived. The psychiatrist Naomi Remen wrote *Kitchen Table Wisdom* to describe the importance of sharing stories for her cancer patients, many of whom were terminally ill (4). "Telling stories is not just a way of passing time," she noted. "It is the way the wisdom gets passed along." Stories are not simply for individual inspiration: they are an essential part of explaining the world. I hope these narratives offer wisdom and provide data to illustrate a policy model. The last portion of this article examines the Heifetz model of adaptive leadership and the challenge for public health leaders in times of social shifts such as the ones these stories describe. I conclude by considering how the wisdom gained from historical analysis and story may lead to change in public policy.

These stories are not meant to be primary historical accounts. Most of the material comes from secondary sources.

## Prologue: The Gilded Age

For a better understanding of health conditions during the early 20th century, it is necessary to understand the economic and social context of the late 1800s. Between the end of the post-Civil War Reconstruction period and the economic panic of 1893, the nation experienced another kind of wrenching civil conflict, that between an agrarian economy and an industrial one. Telephones, electricity, internal combustion engines, and cross-continental railroads all came into use. A laissez-faire economic approach, encouraging unregulated financial power and free market principles, led to accumulation of enormous wealth by "captains of industry": John D. Rockefeller in oil, Andrew Carnegie in steel, and Cornelius Vanderbilt in railroads. The conspicuous consumption and public profiles of the country's most favored citizens led to this era's name, the Gilded Age.

Initially it seemed that the agriculture of the Midwestern states would benefit from the industrial boom. The Homestead Act of 1862 had offered free land for families in the Midwest; 15,000 homestead claims were established by 1865 (5). In the late 19th century, tractors enabled farmers to increase crop size, and railroads carried the harvest to distant markets. But railroad monopolies forced the farmers to accept excessive shipping charges, and farmers went into debt for modern farming equipment to maintain competitive production. A downward economic spiral evolved. Farmers cultivated more land to improve yield, increased yield led to lower crop prices, and farmers plowed even more land to improve productivity. By the 1880s the Midwest had greater per capita debt than any other part of the country (6,7).

In the Northeast, mechanization decreased the requirement for skilled workers and created an enormous number of new jobs for untrained labor. The great northern migration of more than 250,000 African Americans began (8). Still there were not enough workers, and women and children joined the workforce. The number of women in the workforce increased from 2.6 million in 1880 to 7.8 million in 1910 (7,9). By 1910, 25% of US children were in the labor force (10). Still there were not enough, and between 1890 and 1925, 25 million immigrants, first from northwestern Europe and then from southern and eastern Europe, arrived in the United States, many of them settling in New York City (7).

Meanwhile, Southern entrepreneurs yearned to erase the image of a defeated Confederacy and proclaimed "the New South." Henry Grady, editor of *The Atlanta Constitution*, outlined its 3 concepts: north and south were no longer different, black and white were now equal, and industrialization was replacing plantation agriculture (11-13). These themes were echoed in other public forums on the future of the Southern states. The Southern railroad system became one of the best in the country. Cotton mills, coal and iron mines, and tobacco factories became major industries (11). Alongside these factories were old plantations that became tenant and sharecropping lands, where the lives of poor black and white tenant farmers were barely better than those of the earlier slaves.

As the new century neared, the dispossessed fought back. The progressive movement encouraged government activism in immigrant welfare, antitrust policies, women's suffrage, child labor laws, and political corruption (14). The Grange, founded in 1867 in the Midwest, promoted pro-farmer legislation and farming cooperatives. By 1875 it had approximately 850,000 members; it was succeeded by other farmers' alliances that eventually became the core of the Populist political party (6). Samuel Gompers organized the American Federation of Labor (AFL) in 1886 (10). The National Association for the Advancement of Colored People (NAACP), directed by W.E.B. Dubois, formed in 1909 (15).

The Women's Christian Temperance Union (WCTU), established in 1874, had a branch in almost every county in the country by 1883 (16). The organization supported a wide range of social interests, including day care nurseries, homeless missions, prison and labor reform, child labor laws, and women's suffrage. A more secular group, the General Federation of Women's Clubs (GFWC) was organized in 1890 from independent women's clubs (primarily library associations) that already existed across the country (17,18). Both groups' activities developed women's organizational and leadership skills and eventually represented millions of members. Barred from the formal political process but convinced of their moral duty to improve society, these women's groups became a powerful voice in social reform.

The Gilded Age crashed in the Panic of 1893 (19). A run on gold reserves led to a 4-year depression as destructive as that of the 1930s. More than 15,000 businesses went bankrupt; banks, railroads, and farms were particularly hard-hit. The laissez-faire approach had failed to provide economic security for American families.

## Worms

### The story

At the beginning of the 20th century, the Southeast was still recovering from the Civil War. Despite the proclamations of a New South, agriculture remained the primary source of employment, but Southern agricultural yield was the worst in the country. The states' public education and health infrastructures were weak to nonexistent. Twelve percent of white adults and 50% of African American adults were illiterate (20).

The poor of all races still suffered. In the *Plessy v Ferguson* Supreme Court decision of 1896, "separate but equal" accommodations became legal on railroad cars (11), and Southern states extended the interpretation to all public settings, creating a second-class black citizenry with little access to community resources. There was also the grim image of the poor white "cracker," with sallow skin, bare feet, scrawny neck, protuberant abdomen, retarded intelligence, and shiftless habits (20). Poverty existed throughout the South, but a striking band of desperation extended across its coastal plain — a flat, sandy region created by the receding of an ancient sea. Here families lived in shanties without sanitation and supported themselves on subsistence farming (21).

In 1902 the zoologist Charles Stiles discovered that *Necator americanus* — the hookworm, the "germ of laziness" (20) — was widespread in the coastal plain. The hookworm enters its host through the skin, often between the toes, then passes into the bloodstream. It travels through the blood vessels to the lungs, moves up the bronchial tubes to the throat, and is swallowed. The worm then makes its permanent home in the small intestine, where it secretes anticoagulants and sucks blood from the intestinal mucosa of its host. One infected individual may have several hundred parasites that lay up to 10,000 eggs per day; these eggs pass out of the victim's body in the feces. The infection can persist for years; its most striking effect is severe anemia caused by bleeding into the intestinal tract (22). Over time, this chronic blood loss leads to pale skin, weight loss, and abdominal edema; the low blood oxygen levels decrease mental and physical function.

During this same time, the wealthy industrialist John D. Rockefeller turned to philanthropy. He sponsored several projects, including programs in education and agriculture in the South. In 1909 he established the Rockefeller Sanitary Commission for the Eradication of Hookworm Disease. Its purpose was to characterize the extent of hookworm disease in the South and conduct a campaign to reduce its prevalence (20). The first hookworm survey (1910-1911), conducted in 600 Southern counties, identified an infection rate among children of 40% (23) and estimated that 7.5 million Southerners were infected (24). People living in the coastal plain had the highest rates of infection; the sandy soil created the most inviting environment for hookworm larvae. This sandy soil was better for farming than the red clay hills of the Southern piedmont, but agricultural productivity here was the lowest in the region (23,24).

The Rockefeller Commission embarked on an extensive campaign, working closely with state and local partners (20). It promoted physician training and sponsored state laboratories that were equipped to identify hookworm larvae. It encouraged traveling dispensaries that not only diagnosed and treated infected individuals but provided public education in a tent-revival–like atmosphere, using local children's choirs and similar entertainments to encourage attendance. In particular the campaign focused on schools, developing designs for sanitary privies and health education for children and teachers.

The commission stepped around concerns of race. It recognized the importance of black health and education; Rockefeller had funded the black women's seminary that was the precursor to Spelman College in Atlanta, and he had supported other efforts in black education. But Stiles' reports of hookworm in former slaves had created a backlash, as Southern whites blamed African families for bringing the worm to America. The commission's program director did not collect race-specific information from the dispensaries, but he received frequent reports from field observers who informally evaluated the percentage of black participants attending the dispensaries. These were not quantitative assessments, but the commission feared publishing real data would provide fodder for resentful whites who might destroy the project altogether (20).

### The analysis

The science was clear in this event. Hookworm infestation was widespread in the South, and hookworm infection, with its resulting anemia, was a direct cause of sluggish productivity among Southern agricultural workers. Rockefeller's support led to an improved, functional health infrastructure. The challenge was political will.

The New South supporters were incensed by the campaign. The claims of extensive hookworm disease directly countered their efforts to create the image of a prosperous region. Southern newspapers denounced Rockefeller as a carpetbagger and a patronizing Northerner. Some claimed the campaign's recommended prevention measure of wearing shoes was a ruse to create new markets for footwear in the South (20). Perhaps even more critical, the citizens of rural communities often resented and even refused the efforts of outsiders to "control" their schools (20,25).

Despite these challenges, public policy led to change. The commission existed from 1909 to 1914. At its close the campaign had not eradicated hookworm; nevertheless, it left an impressive legacy. The commission was the immediate precursor of the Rockefeller Foundation and its International Health Board, which funded hookworm eradication programs around the world. The campaign enhanced the impetus for reforms already initiated by Progressive Southerners. Rockefeller had previously established a General Education Board to support public education in the South (20). Educators used the hookworm campaign to emphasize that the health of schoolchildren was linked to their learning, leading to programs for school construction, sanitation, and health inspections (25). Accompanying these efforts were increases in school enrollment, attendance, and literacy (23).

The government's recognition of its role in hookworm eradication led to new state and county health departments (20,26). Agricultural productivity also increased between 1910 and 1920, in part because of the improved health of farm laborers (24). Farm demonstration programs, supported by both Rockefeller and the federal Department of Agriculture, proved that proper agricultural techniques allowed successful cotton crops despite the boll weevil. The success of these complementary activities enabled Southern members of Congress to secure passage of the Smith-Lever Agricultural Extension Act of 1914 (23,25), which established the Agricultural Extension Service and provided a combination of federal and state funding to link university agricultural research to local farming practice. This law, considered one of the most important US agricultural bills ever passed, would serve as the model for later legislation in child health.

## Germs

### The story

While Rockefeller promoted privies in the South, other Northern reformists focused their attention on immigrant health in the tenements of urban communities. The enormous waves of immigrants overwhelmed available housing: in New York City, 3 to 4 families might crowd into homes intended for 1. Population density in the tenement sections of the city was the greatest in the world, estimated to reach 290,000 per square mile (27,28). These neighborhoods had no systems for plumbing or sewage disposal; rotting food and horse droppings filled the streets. Only the most low-paying, unskilled jobs were available to these immigrants. The labor unions refused them membership because they had replaced skilled workers. Immigrant women worked outside the home or did piecework; the children wandered the dangerous streets without attention and entered the labor force at an early age (27).

Infant mortality rates in the tenements were the highest in the country. In particular, summer epidemics of cholera infantum, or infant diarrhea, killed thousands of infants every year (29). In New York City in 1882, 50% of infant deaths were attributed to diarrheal diseases (29). Health authorities referred to these as "filth diseases," a concept linked to miasma theories, and attributed the deaths to the filthy conditions and impure air of the urban environment and to the ignorance and poor hygiene of immigrant parents (29).

However, as bacteriology became an established science, contaminated milk was identified as a more specific cause of infant mortality. Pediatricians and social activists agreed that breast milk was the best nutrition for infants, but they also recognized that mothers working outside the home were forced to use artificial feedings (30). Reformers hoped that supplying clean milk to poor immigrant families was an achievable solution to cholera infantum. They turned to the French model of "milk stations": programs in poor neighborhoods where mothers could obtain safe cows' milk for their babies (31).

Philanthropist Nathan Straus, co-owner of the Macy department stores, funded some of New York's first milk stations as well as a pasteurized milk bottling plant; by 1896 these milk stations were feeding more than 2,000 infants a day and supplying more than 600,000 bottles of milk a year (31). The city government established a Division of Child Hygiene within the city health department in 1905; because of the success of the Straus stations, the division provided funds in 1911 to support additional infant milk stations (29). Soon the milk station concept expanded to social and health education programs, including well-child care at station sites and home visiting nurses to educate immigrant families in domestic hygiene and infant care (32).

The Division of Child Hygiene organized after-school programs, "Little Mothers' Leagues," that taught girls how to care for younger siblings while their mothers worked. The girls also received instruction in volunteerism and civics (31). The division's workers hoped the education would enable the first immigrant generation born in the United States to rise out of the poverty that affected their families' welfare.

The measurement of New York infant mortality rates depended on a weak vital record reporting system. However, its data indicate a decline in the infant death rate from 248 per 1,000 infants born in 1885 to 80 per 1,000 in 1919 (29).

### The analysis

In the story of New York infant health, science again provided a solid basis for action. Germs in the milk could be prevented through a city government infrastructure, and political will engendered by the Progressive movement supported such infrastructure. Then individual urban programs grew into a national movement, and over the next 20 years dramatic changes in political will led to frequent shifts in public policy.

The American Association for Study and Prevention of Infant Mortality (AASPIM) formed in 1909. This national organization emphasized that social reform and health education were essential to support healthy children (31). The first priority was to improve vital statistics reporting in all states so infant mortality could be accurately determined. Ultimately, the drive to document infant deaths became a primary force in the development of the modern US vital statistics reporting system (29).

Another plank in the AASPIM platform was to foster communication and coordination among the many organizations involved in infant health. These political efforts led to the establishment of the federal Children's Bureau in 1912, which was mandated to "investigate and report on all matters pertaining to the welfare of children" (33). In turn, the bureau's research into the social aspects of child health supported the call for federal funding to promote local programs, similar to well-baby clinics, which would educate mothers about preventive child care (34). The chief of the Children's Bureau cited the Smith-Lever Agricultural Extension Act as a model for federal funding through matching grants to interested states (29,35).

Bills to support these programs were introduced into Congress in several sessions, but not until 1921 did a bill appear that seemed likely to become law. It faced strong opposition. Politicians argued that it was too expensive and violated the separation of state and federal responsibilities. Physicians were coming into their own as a profession and feared any intrusion into their field; the American Medical Association and American Gynecologic Society claimed that the bill would interfere with the practice of medicine. Opponents of women's suffrage said it would weaken the family and transfer parental authority to the government, and that its premise arose from Bolshevik philosophy (29).

But one political reality above all others favored the bill's passage: women had received the vote in 1920, and women's organizations considered the Sheppard-Towner Maternity and Infancy Act a universal women's issue. A coalition of 14 women's organizations, including the WCTU, GFWC, League of Women Voters, National Council of Jewish Women, and the General Federation of Business and Professional Women, urged members to write their congressmen and senators. *Good Housekeeping*, *McCall's*, and *Ladies' Home Journal* published supporting editorials (29). No one knew whether newly enfranchised women would vote en bloc for women's and children's issues, but no elected official, regardless of personal views, wanted to risk offending an enormous new group of voters. The Sheppard-Towner Act passed in 1921.

By 1922, 41 states elected to match Sheppard-Towner funds. Tens of thousands of mothers and children received prenatal and well-child consultations, visiting nurses made hundreds of thousands of home visits, and educational pamphlets reached millions of readers. Mothers showered the Children's Bureau with letters expressing their appreciation (29). Backers of the act expected easy passage when it was scheduled for renewal in 1926.

However, political will had changed. It was now clear that female voting split along party rather than sex lines. President Coolidge and the Republican party pressed for a reduction in government programs (29). All the opponents of the 1921 passage, and more, appeared in force. Medical practitioners became an even stronger resistant force, and the earlier multidisciplinary partnership of the AASPIM could no longer hold. Only a 2-year extension was granted, and the program permanently closed in 1929. The public policy to support child welfare by integrated health and social programs collapsed.

## Drink

### The story

World War I marked a shift in political winds from Progressivism to a war and postwar conservatism. Among other changes, these new perspectives opened the door for a serious national contemplation of alcohol temperance. Temperance had been discussed for decades: progressive women's groups believed alcohol temperance was a key tool in promoting community welfare (36). Activists blamed alcohol for multiple social evils — crime, violence, unemployment, and poverty. Their focus was a blue-collar laborer who spent his paycheck in the saloon, where he consorted with prostitutes, gamblers, and corrupt politicians, then returned home to thrash his wife and children.

During the war effort, temperance was promoted as a way to reserve grain needed for bread. In addition, "anti-Kaiser" sentiment flourished against the German immigrants who ran most American breweries and controlled most saloons. The Ku Klux Klan, most commonly remembered for its violence against African Americans in the South, also targeted multiple immigrant groups, especially those who were Jewish and Catholic, in the Midwest and Western United States (36,37). The Klan's support of temperance was a reaction to the ritual and social consumption of alcohol by these groups. Temperance came to be associated with Protestant, rural, and conservative voters, while antitemperance was supported by Catholic, urban, freethinking voters. Amid the clash of these groups, the constitutional amendment establishing Prohibition (18th Amendment) passed in 1919.

But alcohol consumption persisted during Prohibition. The spirits of choice depended on social class. The upper class could afford smuggled bonded whiskey from Canada; others turned to homemade moonshine. Some of the poorest citizens drank cheap Jamaican ginger extract. "Jake" was a patent medicine that had been widely available before the 18th Amendment; its designated use was a few drops of extract added to water for treating respiratory infections, indigestion, and irregular menses, but its popularity derived from its 70% alcohol content. The federal Bureau of Prohibition required that the percentage of ginger solids in the extract be raised to 5 g/cm^3^, creating a liquid too bitter to drink for other than medicinal purposes. Regulatory compliance was assessed by boiling down the marketed product and measuring the weight of the remaining solids, so would-be bootleggers had to find adulterants that would reduce the strong taste and meet the required solid weight content (38).

In February 1930, a physician in Oklahoma City, Oklahoma, saw 5 cases in the same day of a mysterious paralysis of the legs (39,40). Investigation by city public health authorities found 65 additional cases. More newspaper reports from across the country accumulated within weeks: Alabama, Mississippi, Georgia, Kentucky, Tennessee, South Carolina, North Carolina, Louisiana, Kansas, Massachusetts, Texas, Missouri, Oklahoma, Ohio — the count of cases grew to the tens of thousands (38). The characteristic findings were initial numbness in the legs, followed by a paralysis of the toes and feet that caused a "foot-drop" gait in which victims lifted their feet high and then put down the toe before the heel. This high-stepping, foot-slapping gait became known as the "jake walk." Often a similar wrist drop paralysis followed (40).

Recovered samples of the extract went to the Bureau of Industrial Alcohol, which identified the adulterant as tri-ortho-cresyl phosphate (TOCP), a chemical that at the time was not recognized as poison. The National Institute of Health conducted field investigations and collected more samples. Additional pharmacologic and animal testing indicated that the suspected samples were contaminated with TOCP and that this compound caused the jake paralysis (40,41).

### The analysis

The infrastructure of laws and bureaucracy begins this story. Individual efforts to subvert the infrastructure led to catastrophe, which in turn led to scientific efforts to identify a poison. But political will generated only a weak response.

Two partners of a Boston firm were charged with illegal alcohol distribution and falsely labeled products and received fines and probation. In 1932, one eventually began a 2-year prison term (40). The Food and Drug Administration was not yet empowered to require premarket testing of products for safety (42). Thus, the conspirators could not be charged for injury to thousands of consumers.

Nor did public policy change. The victims of jake paralysis were marked by their unique gait; conservative neighbors and even the afflicted themselves believed their own irresponsibility had destroyed their lives. Few of those who were poisoned made a full recovery: the United Victims of Ginger Paralysis Association claimed 30,000 members and sought federal compensation for those injured, without success (40).

## Dementia

### The story

The Mississippi River wanders for more than 2,000 miles through the middle of the country, draining 41% of the continental United States (43,44). For centuries the river has brought fertile soil down its course and deposited it in the Delta region of Mississippi and other nearby Southern states. But these nutrients traveled with floodwaters that periodically destroyed structures and crops and drowned people and livestock. With industrialization, the need to prevent this flooding grew urgent.

Inevitably, politics controlled river development. One-crop cotton farming still dominated the lower river basin after the Civil War. The plantation owners manipulated taxation at local levels, exploited the cheap labor of ex-slaves, and obtained taxpayer assistance at state and federal levels to maintain river flow. In the 1880s the river was channeled to allow shipping. In 1884 the Mississippi legislature created the Yazoo-Mississippi River Levee District to build levees along state tributaries of the Mississippi River, and in 1917 the Army Corps of Engineers took over levee building (43,44). It was to all players' advantage, except that of the sharecroppers, to believe that inexpensive levees (the "levee-only policy") were sufficient protection against floods.

The 1-crop farming system linked the Delta region's diet to the seasonal economics of cotton: salt pork, corn meal, and cane syrup were staples, while vegetables, milk, eggs, and fresh meat seldom appeared on the dinner table of a sharecropping family, especially in the winter. Pellagra was widespread. The initial symptom, a "butterfly" dermatitis that spread across the face and other sun-exposed parts of the body, created the red-neck stigmata that popular national culture extended to the South as a whole, though the rash was by no means limited to those of light skin. Diarrhea soon followed, and when the afflicted grew demented their family members knew that death might come soon (mortality rates at this stage were 50% and higher), completing the "4 *D*'s" of the pellagra syndrome (45).

By 1920 Joseph Goldberger had made his name from his pellagra research in southern orphanages, "insane asylums," and mill towns (45). He identified that pellagra resulted from the "meat, meal, and molasses" plate and was a dietary deficiency disease rather than the result of infection or improperly milled corn. Field research in pellagra progressed from mill towns to the Georgia State Sanitarium in Milledgeville, where victims of pellagra dementia were housed. Goldberger's work confirmed that pellagra was common in the South. The diet investigations identified brewer's yeast as an effective, inexpensive preventive and cure (45).

Henry Grady was long dead, but other New South spokesmen were infuriated. Once more Yankee voices had proclaimed a widespread disease of poverty in the South. Yet the Mississippi state board of health recognized the pellagra epidemic and in 1920 held a state conference to discuss action. In 1921 an investigation of the Delta region found it had 60% of the state's cases but only 20% of the state's population (45).

Then came what Herbert Hoover described as the "greatest disaster in peace times in our history": the 1927 Mississippi flood (45). The river ignored the levees and flooded 23,000 square miles; by early June, 112 counties and 12 states were under water, and 700,000 people were displaced (45). The first pellagra cases in the Red Cross camps were reported in July.

Goldberger toured Tennessee, Mississippi, Arkansas, and Louisiana and estimated that 45,000 to 50,000 cases existed across the region. The Red Cross created a massive, uncontrolled natural experiment, distributing 12,000 pounds of yeast. The distribution was followed by a rapid decline in the number of reported cases and improvement in disease symptoms. For the first time, the pellagra-prevention diet proved effective in a large population (45).

### The analysis

Scientific theory was confirmed on a grand scale. Then the waters receded. The displaced went home, and infrastructure development began. Pellagra education had begun in the camps; now the Red Cross initiated long-term prevention with additional information, food packages, even cows sent home with departing families. Farmers were advised to set aside land for a food crop as well as an economic crop. The state health department of Mississippi launched a major education campaign, distributing yeast through local drugstores and providing weekly clinics (45).

Yet the disease didn't disappear. In fact, it increased during the next 2 years as rural sharecroppers' diets remained unchanged — poverty and culture barring the way to healthful foods. Public policy was stalled by old political will and economics. In 1927 Goldberger angered Southerners yet again with an article in *Public Health Reports*: he identified 1-crop farming as the underlying cause for the Mississippi epidemic (46). But the disease persisted until commercially produced bread was fortified with vitamins in the late 1930s and 1940s (47).

## Conclusion

### Public health leadership

We can look across these stories as well as within them and see elements familiar to modern public health professionals: a shifting economic base, global human migrations, changing avenues of communication, clashing cultures, prejudice against "the other," the aftereffects of war, the belief that the disadvantaged deserve their ill luck. The Richmond-Kotelchuck model offers the analytic structure that demonstrates that these elements are all intertwined with health. Public health workers have had mixed success in addressing the barriers they identify. Our science works diligently on the risk factors, causes, diagnoses, and treatments of disease. The initial efforts may not succeed, but the skill and impetus to continue searching often result in ultimate success. Public health experts excel in building infrastructure. In rural or urban settings, for children or adults, in the mainstream population or for the disadvantaged, addressing the need for immunization or chronic health problems such as hypertension, public health programs have designed unique infrastructures to support populations in need.

But these 4 stories bring us back to political will. Ronald Heifetz, author of *Leadership Without Easy Answers*, suggests 2 kinds of leadership problems (48). The first requires *technical* solutions: the problem is known, the solution is recognized, and to move from one to the other requires the use of known skills and experience. Civic disruption is no more than mild, and social attitudes remain much the same. The "Back to Sleep" campaign to prevent sudden infant death syndrome (SIDS) might be an example of successful technical problem solving. Research identified a major risk factor for SIDS, a national public education campaign ensued, and parents found it easy to put babies on their backs instead of stomachs to sleep (49).

The second leadership problem requires *adaptive* change. Adaptive change is about political will. The change requires a significant paradigm shift in science, systems, and social attitudes, and the shift must occur in the face of substantial resistance from the affected sectors. Leaders may take the easier course of treating adaptive challenges as technical ones — people accept that professionals will provide technical answers, while no one may know the answer to or consequence of an adaptive change.

### Adaptive change

The need for paradigm shift exists when society's expressed values and its behavior differ. The Progressive Era arose from the public realization that laissez-faire economics and industrial power did not provide the individual economic security they claimed. The formations of the NAACP, the Grange, the AFL, and the GFWC marked the beginnings of a shift from laissez-faire economics to new expectations of government. The shift carried upheaval — women and blacks demanding social change, labor power rising to balance the employers', agricultural systems giving way to industrial ones. New stories appear when old ones are not sustainable; they are resisted because they arrive with economic and social costs.

An adaptive need may be recognized without receiving action. Because change is unpredictable, only a sense of urgency will drive people to consider it. If pandemic influenza is sweeping the country, high levels of public distress will create the urgency for change. At other times the public refuses to accept a problem as urgent. The New South boosters, the industrialists, and the anti-immigration movement were not ready to accept that their stories were inadequate. In the early days of the recognition of acquired immune deficiency disease, most of the population believed the infection was restricted to a small number of men having sex with other men. Popular attitudes were similar to those toward the victims of jake paralysis: the affected individuals' behaviors were socially unacceptable, therefore they deserved their affliction and "normal people" need not worry.

As stress increases, the temptation rises to find easier alternatives. Women leaders of the temperance movement believed outlawing alcohol would eliminate multiple social ills. The fight for temperance was long and difficult, but it was still easier to pass prohibition legislation than to control violence, prostitution, or poverty. The current debate about universal health care is a modern example. Over the past 2 decades provider-driven care, health maintenance organizations, health savings accounts, consumer-controlled health plans, government-based insurance, tax credits, and a host of other technical options have been proposed. The adaptive question, however, is whether our social contract includes providing health care for every citizen. Until we recognize the question, technical solutions will not succeed.

Heifetz notes that the final, and perhaps most difficult, leadership role is giving the challenge back to the citizens. Leaders are tempted to "claim" big issues, while society waits for them to solve the problems and people go about their usual lives without accepting their essential roles in solutions. Prohibition did not prevent alcohol consumption, much less its associated social evils, because much of the population did not support its premise. Authority can direct attention to the challenges; it is not able to resolve adaptive problems without involving the populace.

### Problem-solving for the 21st century

One of the greatest risks for public health professionals is to accept ownership of a problem. We recognize the importance of building partnerships, crossing boundaries, and involving communities, but we are not yet sufficiently humble about our ability to create adaptive change. We are strong in technical solutions, but how skilled are we at affecting the third sector of the Richmond-Kotelchuck model? We know that scientific evidence and infrastructure related to immunizations, safe sex, regular exercise, tobacco avoidance, and even universal health care are not always sufficient to drive political will. Do we have the ability to change social context? And can we gain wisdom from stories of the past?

Now, in the beginning of the 21st century, societal pressures are building once more. In the United States another major economic transition is progressing, changing from an industrial base to a service-oriented, information base. Accompanying that change are labor migrations associated with a global economy; this time work is traveling abroad to the workers. At the same time, immigrants continue to arrive, and anti-immigrant bias is now directed toward those with Mexican and South American origins. The quality of child health programs and public schools is at risk. The percentage of older adults in the population is rising: a greater Medicare burden is predicted, but the generation now maturing will be healthier and live longer. What will this mean for the national workforce and health care demands? Shifting warfare, infrastructure, and ecology concerns overlay these other stresses.

How do these stories speak to the role of public health workers? Perhaps we cannot force change, but we can offer leadership in our own arena, we can inform paradigm shift, we can tip events toward a successful resolution. The Rockefeller Commission and Southern Progressives combined an understanding of hookworm infestation, agricultural productivity, and school health to stimulate political will for better lives among rural Southerners. Goldberger's understanding of social constructs informed his research and recommendations for eradicating pellagra, and his work was essential, ultimately, for the fortification of bread. Sometimes our success is partial. Child health activists and the Children's Bureau placed an early emphasis on the connection between health and social welfare; later medical interests separated those concepts. Sometimes we fail. Public health researchers identified the cause of jake paralysis, but there was no call to improve the lives of the afflicted or to find ways to prevent future catastrophe.

At its heart, public policy is about the choices of citizens in a cooperative endeavor. What are the early warning signs that these choices are at odds with professed beliefs? Where might we hear the first rumblings of a social shift? Will we understand the archetypal stories people use in health and policy decisions? Perhaps we need to teach ourselves another way of observing, to learn more about incorporating intuitive as well as data-driven evidence into action. Too often, of course, we have acted without evidence, and no one advocates a return to that practice. But given our struggles with political will, it seems we need not only new data but also new tools for understanding the data.

The events described here grew out of the earlier history of the Gilded Age and in turn created the context for what we face today. The stories illustrate that public policy results from multiple elements, some of which developed long before current crises. In helping us understand political will, the past offers not only informative case studies but also narrative components of contemporary challenges. For us to offer leadership to influence change, we need to immerse ourselves in story, past and present.
